# Correction: Natarajan et al. Noise-Induced Hearing Loss. *J. Clin. Med.* 2023, *12*, 2347

**DOI:** 10.3390/jcm13040944

**Published:** 2024-02-07

**Authors:** Nirvikalpa Natarajan, Shelley Batts, Konstantina M. Stankovic

**Affiliations:** 1 Department of Otolaryngology-Head and Neck Surgery, Stanford University School of Medicine, Palo Alto, CA 94304, USA; 2 Department of Neurosurgery, Stanford University School of Medicine, Palo Alto, CA 94304, USA; 3 Wu Tsai Neuroscience Institute, Stanford University, Stanford, CA 94305, USA

**Error in Table**

In the original publication [1], there was a mistake in Table 1 as published. The permissible noise exposure times by OSHA were in error. The corrected Table 1 appears below. The authors state that the scientific conclusions are unaffected. This correction was approved by the Academic Editor. The original publication has also been updated.

**Table Legend**

The wording to describe the unit adjustment and references needed updating to reflect corrections to Table 1. The correct legend appears below.

“The NIOSH limits represent clinically safe levels, and the OSHA limits are more liberal to account for practical issues in industry [204,205]. Units of time are standardized for ease of comparison. Abbreviations: dB, decibel; OSHA, Occupational Safety and Health Administration; NIOSH, National Institute for Occupational Safety and Health.”

The authors state that the scientific conclusions are unaffected. This correction was approved by the Academic Editor. The original publication has also been updated.

**References**

Two references were updated to support the revised Table 1 (#204, 205).

204. OSHA. 1910.95 App A—Noise Exposure Computation. Available online: https://www.osha.gov/laws-regs/regulations/standardnumber/1910/1910.95AppA (accessed on 27 March 2023).
205. NIOSH. Occupational Noise Exposure—Revised Criteria 1998. Available online: https://www.cdc.gov/niosh/docs/98-126/ (accessed on 27 March 2023).

One reference (#206) was updated to fix a broken link since publication.

206. Centers for Disease Control and Prevention. Noise and Occupational Hearing Loss. Available online: https://www.cdc.gov/niosh/topics/noise/noise.html (accessed on 27 March 2023).

With these corrections, the order of the references does not change. The authors state that the scientific conclusions are unaffected. This correction was approved by the Academic Editor. The original publication has also been updated.

## Figures and Tables

**Table 1:** Occupational noise exposure limits recommended by NIOSH and OSHA.

Sound Pressure Level (dB)	Permissible Exposure Time	
	NIOSH	OSHA
120	9 s	7 min 30 s
115	28 s	15 min
112	56 s	22 min 48 s
110	1 min 29 s	30 min
109	1 min 53 s	34 min 12 s
106	3 min 45 s	52 min 12 s
105	4 min 43 s	1 h
103	7 min 30 s	1 h 18 min
100	15 min	2 h
97	30 min	3 h
95	47 min 37 s	4 h
94	1 h	4 h 36 min
91	2 h	7 h
90	2 h 31 min	8 h
88	4 h	10 h 36 min
85	8 h	16 h
82	16 h	24 h 18 min
81	20 h 10 min	27 h 54 min
80	25 h 24 min	32 h
