# Supplementary material for: Noise-Induced Hearing Loss
Source: J Clin Med. 2023 Mar 17;12(6):2347. doi: 10.3390/jcm12062347 (PMC10059082; doi:10.3390/jcm12062347)
Supplement: Supplementary file 1 [file jcm-12-02347-s001.zip › jcm-2237521-supplementary.pdf]

SUPPLEMENTAL MATERIAL *for* Noise-Induced Hearing Loss

Table S1. Clinical trials of interventions for NIHL registered at ClinicalTrials.gov without reported results

ClinicalTrial.gov ID/Name	Year registered	Status	Intervention	Study design	Population, N	Active arm, N	Comparator arm, N	Primary outcome	Secondary outcomes	Sponsor
NCT05511753: The effect of acupuncture on patients with noise-induced hearing loss	2022	Not yet recruiting	Electro-acupuncture	Open label interventional study	Adults (20-65 y) with NIHL, N=80	Acupuncture for 15 min three times/week for 6 weeks.	Conventional treatment (not specified)	Change in PTA at 2, 4, and 8 kHz from baseline	None	China Medical University Hospital
NCT05086276: FX-322 in adults with acquired sensorineural hearing loss	2021	Active not recruiting	Intratympanic injection of FX-322	Phase 3 double-blind RCT	Adults with NIHL or idiopathic sudden SNHL, N=124	1 dose FX-322, n=62	1 dose placebo, n=62	Change in speech perception using WR tests, Day 90 from baseline	Change in PTA, tinnitus assessments, other patient report surveys	Frequency Therapeutics
NCT04766853: Verification of the efficacy/safety of the intratympanic drug delivery for hearing loss	2021	Recruiting	Intratympanic steroid delivery for hearing loss	Randomized, double-blind study	Adults (19+ y) with >25 dB hearing loss on PTA, N=26	Dexamethasone + hyaluronic acid	Dexamethasone + saline	Healing of tympanic membrane 3-4 weeks post-injection, duration of drug and inflammation in middle ear with CT 1 day or week post-injection, evaluation of hearing threshold with PTA 3-4 weeks post-injection	None	Seoul National University Hospital

NCT04768569 / Noise-Induced Hearing Loss-Acute Exposure Treatment (PINIHL-AET)	2021	Enrolling by invitation	Zonisamide	Phase 1 double-blind RCT	Adults scheduled for skull-based surgery that requires ≥1 h of drilling, N=180	100 mg zonisamide pre-op + placebo post-op, or placebo pre-op + 100 mg zonisamide post-op, n=60 each	Placebo pre-and post-op, n=60	Ratio of PTS-positive subjects in 30 days post-op (≥10 dB hearing loss at any frequency from 2-6 kHz)	None	Washington University School of Medicine
NCT04774250 / Noise-Induced Hearing Loss-Acute Exposure Treatment (UA) (PINIHL-AET)	2021	Enrolling by invitation	Zonisamide	Phase 2 double-blind RCT	Adult police officers scheduled for firearm training and/or certification, N=126	100 mg zonisamide, n=63	Placebo, n=63	Ratio of PTS-positive subjects ≥10 dB shift at ~30 days after training	Change from baseline in DPOAE, ultra-high frequency audiometry, electrocochleography, WIN. Phenotypic genetic analysis	Washington University School of Medicine
NCT04482998: Efficacy of Various treatments for acute acoustic trauma	2020	Unknown	Early or delayed steroids with or without HBOT	Retrospective cohort study	Israeli Defense Force members with AAT, N=250	Early (within 7 days of AAT) or late (after 7 days) delivery of steroids only, HBOT + steroids, or HBOT	No treatment	Hearing improvement until 1 year of AAT	Change in tinnitus and OAE until 1 year after AAT	Medical Corps, Israel Defense Force
NCT04601909: FX-322 in Adults With Age-Related Sensorineural Hearing Loss	2020	Completed	Intratympanic FX-322	Phase 1b double-blind RCT	Adults with age-related SNHL, NIHL, or sudden hearing loss, N=30	1 dose FX-322, n=24	1 dose placebo, n=6	Systemic and local safety, suicide risk assessment 3 months post-injection	PTA, WR, WIN, and tinnitus assessments 3 months post-injection	Frequency Therapeutics

NCT03878875: Does Sound Conditioning Protect Against Temporary Hearing Damage	2019	Completed	Sound conditioning (low level noise) before loud music exposure	Observational cohort study	Adults (18-35 y) with normal hearing, with or without prior loud music exposure, N=40	10 min low level noise before loud recreational music exposure in subjects with prior history of high exposure, n=20	10 min low level noise before loud recreational music exposure in subjects with history of low exposure, n=20	Change in PTA, DPOAE, TFI, MEMR, SIN 1 day and 1 week post-experimental exposure	None	Imperial College Healthcare NHS Trust
NCT03834714: Noise Exposure and Near-Infrared Light	2019	Completed	NIR light therapy (Earlight Generation 1.4 device)	Randomized, double-blind crossover study	Adults (18-45 y) with normal hearing, N=14	4 sessions noise stimulus and 2 sessions NIR light	4 sessions noise stimulus and 2 sessions sham	Change from baseline in air conduction threshold (500-8000 Hz)	Change in OAE and central auditory performance from baseline	University of Miami
NCT04120116: FX-322 in Adults With Stable Sensorineural Hearing Loss	2019	Completed	Intratympanic administration of FX-322	Phase 2a double-blind RCT	Adults (18-65 y) with stable NIHL or sudden SNHL, N=95	1, 2, or 4 doses FX-322	4 doses placebo	Change from baseline in speech intelligibility, WIN, PTA at Day 210 post-injection. Safety of injection	Change in extended high frequency PTA, surveys of tinnitus and hearing ability	Frequency Therapeutics
NCT02779192: A Phase 2b Study of SPI-1005 to Prevent Acute Noise Induced Hearing Loss (PANIHL)	2016	Unknown	SPI-1005 (ebselen) prior to sound challenge	Phase 2b double-blind RCT	Adults (18-50 y) with history of noise exposure, N=180	Oral 200 or 400 mg ebselen, twice daily, 7 days	Twice daily placebo	Reduction in the incidence of a significant PTA shift, 1 day post-sound challenge from baseline	Improvement in WR score within 1 day post sound challenge	Sound Pharmaceuticals, Inc.
NCT02259595: Study to Determine the Safety, Tolerability, and Pharmaco-kinetic Profile of HPN-07	2014	Completed	HPN-07 or HPN-07 + NAC	Phase 1 double-blind RCT	Healthy adults (18-55 y), N=32	1 dose HPN-07 maximum tolerated dose plus NAC 120 mg	1 dose HPN-07 500, 1000, or 1500 mg plus placebo	Safety and tolerability of HPN-07, alone and in co-administration with NAC	Area under the curve, maximum plasma concentration, half-life, volume of distribution, mean transit time, total	Otologic Pharmaceutics, Inc.

and HPN-07 Plus NAC									clearance at various intervals post-dosing	
NCT02257983 : Protective Effects of EPI-743 on Noise-Induced Hearing Loss	2014	Completed	EPI-743 prior to loud noise exposure	Phase 2a double-blind RCT	Healthy adults (18-30 y), N=77	9 days twice-daily EPI-743 400 mg, noise exposure on Day 8	9 days placebo, noise exposure on Day 8	Change in PTA at Day 9 from baseline	Time to recovery following acute noise exposure on Day 9	Edison Pharmaceuticals, Inc.
NCT02049073 : Prevention of Noise-induced Hearing Loss	2014	Withdrawn	Zonisamide or methyl-prednisolone prior to loud music exposure	Open label parallel assignment study	Healthy adults (18-30 y), N not stated	Oral zonisamide 100 or 200 mg daily for 2 weeks or as 1 dose; or 1 dose oral methyl-prednisolone 32 or 64 mg	no medication	PTA at 2,3,4 and 6 kHz 15 minutes post-4 h music exposure	DPOAE and extended intervals of PTA testing	Washington University School of Medicine
NCT01727492 : Prevention of noise induced damage by use of antioxidants	2012	Unknown	NAC and Mg prior to leisure noise	Randomized double-blind placebo-controlled cross-over study	Adults (18-25 y) with temporary tinnitus after noise exposure: loudness >5 on a VAS, N=30	600 mg NAC and 200 mg Mg	Placebo	50% decrease in tinnitus loudness scored on VAS vs. placebo	. 50% decrease in tinnitus duration scored on VAS and decrease in TTS vs. placebo	University Hospital, Antwerp
NCT00802425 / Efficacy of AM-111 in Patients With Acute Sensorineural Hearing Loss	2008	Completed	Intratympanic injection of AM-111	Phase 2 double-blind RCT	Adults (18-60 y) with unilateral acute SNHL (including noise) with onset ≤48 h, N=210	Single dose intratympanic AM-111 high or low dose (dose not specified)	Single dose intratympanic placebo	Change in hearing loss in dB from baseline and Day 7	Change in hearing loss in dB from baseline and Days 3, 30, 90	Auris Medical AG

See Table 2 for registered completed trials that have reported results. Abbreviations: Abbreviations: AAT, acute acoustic trauma; CT, computed tomography; dB, decibel; DPOAE, distortion product otoacoustic emissions; h, hour; HBOT, hyperbaric oxygen therapy; kHz, kilohertz; MEMR, middle ear muscle reflex; Mg, magnesium; min, minutes; NAC, N-acetylcysteine; NIHL, noise-induced hearing loss; NIR, near infrared; op, operation; PTA, pure tone audiometry; RCT, randomized controlled trial; SIN, speech in noise; SNHL, sensorineural hearing loss; TFI, Tinnitus Functional Index; TTS, temporary threshold shift; VAS, Visual Analog Scale; WIN, words in noise test; WR, word recognition; y, year.
